# Acute Toxicity of Commercial Wildfire Retardants to Two Daphniid Species (*Ceriodaphnia dubia* and *Daphnia magna*)

**DOI:** 10.3390/toxics12080548

**Published:** 2024-07-29

**Authors:** Darlan Quinta Brito, Carlos Henke-Oliveira, Eduardo Cyrino Oliveira-Filho

**Affiliations:** 1 Faculty UnB at Planaltina, University of Brasília, Brasilia 73345-010, DF, Brazil; darlanbrito@unb.br; 2 Department of Ecology, University of Brasília, Brasilia 70910-900, DF, Brazil; carloshenke@unb.br; 3 Embrapa Cerrados, Laboratory of Ecotoxicology, Road BR-020, km 18, Planaltina 73310-970, DF, Brazil

**Keywords:** fire retardants, freshwater, acute assays, microcrustaceans

## Abstract

In the face of global climate change, there has been an increase in wildfires around the world, highlighting the need for improved firefighting techniques, such as the use of fire retardants (FRs). These products can enter aquatic systems directly or through runoff, posing potential risks to aquatic biota. In this study, the acute toxicity (24-h/48-h EC50) of three distinct FRs (N-Borate, N-Phosphate+, and N-Phosphate−) was assessed on the immobility of freshwater microcrustaceans *Ceriodaphnia dubia* and *Daphnia magna*. The toxicity of the FRs varied up to two orders of magnitude, all of which presented risks to cladocerans even at dilutions much below those recommended by their manufacturers. Among the tested FRs, N-Phosphate− emerged as the most harmful to both species. Specifically, for *C. dubia*, the 24 h EC50 was 0.005% and the 48 h EC50 was 0.0019%, while for *D. magna*, 24 h EC50 was 0.003% and the 48 h EC50 was 0.0023%. With the increasing use of FRs for wildfire control, our study highlights the toxicity of newly formulated FRs to daphniid species and emphasizes the need for further evidence-based evaluations of their effects on freshwater ecosystems, which is crucial for choosing FRs that pose the lowest hazard to zooplankton communities.

## 1. Introduction

Fires significantly shape ecosystems, particularly savannas, and have considerable cultural, social, and economic importance [1]. Climate change has led to more frequent and longer-lasting wildfire seasons and to extended dry periods [2,3,4]. Megafires have severely affected ecosystem services by reducing water and soil quality, degrading habitats and biodiversity, impacting carbon cycling, and causing substantial economic losses through damage to infrastructure, agriculture, and forestry [5].

Wildfires are commonly associated with land-use changes in Brazil—like deforestation and land clearance for agriculture—and with conditions of high temperatures, little rainfall, and low humidity [6]. They have also arisen from large amounts of fuel load over the landscapes previously shielded from fire regimes [7].

Brazil presents the highest frequency of fires in South America [1], with large annual fire episodes historically associated with high deforestation rates [8]. These fires have historically affected vast areas across Brazilian biomes, including the Cerrado—a neotropical savanna where fire plays a crucial ecological role—the Amazon, and the Pantanal [9].

The impacts of wildfires on aquatic environments are typically short-lived and involve the sudden input of large quantities of ashes, leading to a sharp rise in chemical concentrations within the water column and causing harmful effects on aquatic life [10,11,12]. Extreme fires are expected to become yet more prevalent due to climate change [5,13], and this underscores the need for implementing fire management strategies to mitigate their environmental, public health, and economic impacts [8,14].

Over the past few decades, advancements in firefighting strategies have introduced more effective tools for combating wildfires, including the deployment of fire retardants [15]. Fire retardants (FRs) are formulated from a blend of chemicals mixed with water to efficiently control and extinguish fire [16]. These agents, which include foams and gels, assist in cooling the fuel, suppressing vapors, and inhibiting any potential re-ignition [17].

The application of fire retardants (FRs), either through ground or aerial methods, may cause them to end up in aquatic environments, either by direct application or via runoff [18,19]. Studies have reported the adverse effects of FRs, including mortality in fish [20,21], hazardous implications for algae [22], zooplankton [23] and the structure of the aquatic community [24], thus highlighting the toxic nature of these chemicals in surface waters.

Despite the absence of national and regional regulations for these chemicals in Brazil [15], FRs have been used in firefighting efforts, notably in a megafire which consumed one third of the Pantanal biome [25].

Previous studies that investigated the toxicity of FRs are outdated and lack ecotoxicological data on the latest formulations [22,26]. Given the multiple pathways through which contamination can occur, it is essential to evaluate the toxicity of FRs to understand their impact on aquatic organisms due to the complexity of their chemical mixtures. Moreover, the literature reveals a lack of information on the toxicity of available brands of FR by manufacturers, particularly the newer products purported to be non-toxic to freshwater organisms. In this respect, we assessed the acute toxicity of three commercially available FR brands to the freshwater microcrustacean (Cladocera, Crustacea) *Ceriodaphnia dubia* (Richard, 1894) and *Daphnia magna* (Straus, 1820).

## 2. Materials and Methods

### 2.1. Fire Retardant (FR) Formulations

The FRs chosen for this study are strongly advertised by their manufacturers for use in extensive firefighting operations. The three commercial formulations evaluated are proprietary blends, with their chemical components revealed either on the product labels or in the data sheets (Table 1). The application concentration varies according to the type of fire being addressed, as recommended by the manufacturers. For this study, the concentrations applied were those recommended for wildfires (Table 1).

### 2.2. Chemical Analysis

The chemical composition of the FRs was obtained via acid extraction, and the determination of metals was achieved via Atomic Emission Spectrometry (ICP-AES—Optima 8300 DV Perkin Elmer, Waltham, MA, USA), in accordance with an EMBRAPA 1997 protocol [27]. The elements analyzed included Aluminium (Al), Boron (B), Calcium (Ca), Iron (Fe), Potassium (K), Magnesium (Mg), Sulphur (S), and Phosphorous (P). The total N contents were determined using the semimicro Kjeldahl method [27]. Chemical analysis of the samples was accomplished by acid extraction (HClO_4_:HNO_3_ 4:1). Standards ranging from 0.5 to 40 mg/L (Merck Certified Solutions at 1000 mg/L) were used for analytic quality assurance of the analyzed elements, with a Limit of Detection (LoD) set at 0.001 mg/L.

These elements were selected due to their common presence as active ingredients in FR formulas (Table 1). The specific products from manufacturers were not directly named; instead, identification was based on the dominant compounds in each tested FR (Table 1). For this study, we used samples of three commercial FRs, as follows: N-Borate FR (characterized by dominant levels of N and B), N-Phosphate+ FR (noted for high levels of N and P), and N-Phosphate− FR (marked by predominant N and P levels, but at lower concentrations). These samples were supplied by the manufacturers and/or firefighting staff.

### 2.3. The Solubilization of FRs

The solution was prepared by mixing the FRs with distilled water at the manufacturer-recommended application rate (volume/volume ratio). Samples were then taken from this mixture for the analysis of water-soluble elements, using a concentration that was one-tenth of the proportions recommended by the manufacturers. The samples of the FR solution were passed through a cellulose ester filter with a pore size of 0.45 μm and then stored at 5 °C in a freezer for analysis of water-soluble elements using ion chromatography techniques (Compact IC 761 Metrohm). This method was applied to detect cations, including Lithium (Li^+^), Sodium (Na^+^), Ammonium (NH_4_^+^), Potassium (K^+^), Calcium (Ca^2+^), and Magnesium (Mg^2+^) and anions, including Fluoride (F^−^), Chloride (Cl^−^), Nitrate (NO_3_^−^), Nitrite (NO_2_^−^), Bromide (Br^−^), Phosphate (PO_4_^3−^), and Sulphate (SO_4_^2−^) (Table 2 and Table 3) [27]. The detection limits for both cations and anions were set at 0.001 mg/L, and standard ion chromatography ranges from 0.02 to 50 mg/L, based on Merck certified solutions (1000 mg/L).

To assess the quality of water, physicochemical parameters were evaluated. Dissolved oxygen (DO) levels, pH, and electrical conductivity (EC) were analyzed using an AK 88 (AKSO, São Leopoldo, RS, Brazil) multiparameter device.

### 2.4. Maintenance of Test Organisms

Microcrustaceans, including *C. dubia* and *D. magna,* are notably sensitive to pollutants, genetically stable, and consistently available throughout the year. These characteristics render them useful bioindicator species for environmental monitoring [28]. In our study, they served as the test organisms to evaluate the impact of FRs on invertebrate species in the pelagic zone.

For the assays, neonates of *C. dubia* and *D. magna* (<24-h old) were utilized. The *C. dubia* cultures were maintained in a laboratory setting under controlled conditions: temperature was maintained at 25 ± 2 °C, light intensity ranged from 500 to 1000 lux, and they were subjected to a 16:8 h light/dark cycle. *D. magna* cultures were kept at an incubator temperature of 20 °C (±2). Both types of cultures were stored in 500-mL glass containers filled with culture medium, with each new batch starting with 30 individuals. The cultures received maintenance on a weekly schedule and were fed daily with the green microalgae *Raphidocelis subcapitata*, at an approximate density of 10^5^ cells/mL, in addition to an organic extract (fish food). The methodology was in accordance with Brazilian Technical Standard Methods NBR 13373: 2010 [29,30].

### 2.5. Preparation of the Test Solution

The three FRs evaluated were in liquid form. To prepare stock solutions, they were diluted according to the recommended ratios in a culture medium appropriate to each test species. For *C. dubia*, the culture medium had a pH of 7.2 ± 0.2 and was made of synthetic soft water with a hardness of 40–48 mg/L. For *D. magna*, the medium used was M4, with a hardness of 175–225 mg/L as CaCO_3_, aligning with standard procedures in ecotoxicological testing [30]. Nominal concentrations of the FRs were expressed as percentages, calculated by dividing the FR volume by the total solution volume prepared in the culture medium, reflecting the convention used in manufacturers’ labels and by firefighting professionals.

Test dilutions were made by diluting the stock solution with the culture medium. The specific dilution levels used in the toxicity tests (Table S1) were established based on preliminary laboratory tests, starting with an initial dilution ratio of 10. These dilutions were then adjusted to smaller dilution factors, with particular attention to those dilutions where effects were observed in 50% of the organisms (EC50).

### 2.6. Acute Toxicity Tests

Acute toxicity tests on *C. dubia* and *D. magna* were conducted in accordance with the OECD 202 guidelines [31], incorporating modifications for these species as outlined in the ABNT/NBR 13373 technical standard [29]. The endpoint for these tests was determined by the immobility rate (lack of movement following agitation) after 24 h and 48 h of exposure. Beakers of 30 mL were used as assay recipients, with a volume of 15 mL of gross sample. Each beaker contained five organisms, with three replicates for each dilution level and the control (Table S1). Test beakers were covered with a clear plastic sheet to minimize water loss by evaporation during the test. The exposure of the species was not renewed throughout the test to mimic a single application, which is most relevant in the context of applying FRs in firefighting. 

The toxicity data were presented as EC50 values, representing the median concentration required to observe an effect in 50% of the exposed organisms. The EC50 is an inversely proportional measure; thus, lower EC50 values indicate higher toxicity of the tested compound. Statistical analyses followed the Brazilian ABNT standard methods, with EC50 values determined through non-linear regression analysis values and a 95% confidence interval (*p* < 0.05), using logarithmically transformed test concentrations and a four-parameter logistic equation. All analyses were performed using GraphPad Prism software version 9.0.

## 3. Results

### 3.1. Chemical and Solubilized Compounds of FRs

Chemical analysis of the three FRs revealed a varied composition of elements. In N-Borate FR, (N) and (B) were the primary elements, with concentrations of 2662 mg/L and 1953 mg/L, respectively. N-Phosphate+ FR showed high levels of (N) at 2940 mg/L and (P) at 2937 mg/L, along with notable quantities of (Fe) at 427 mg/L, (S) at 229 mg/L, and (Al) at 98 mg/L. Conversely, N-Phosphate− FR had lower concentrations of N and P, at 507 mg/L and 621 mg/L respectively, one-sixth of those in N-Phosphate+ FR. Of the three FRs, N-Borate FR had the highest concentration of (B), at 1953 mg/L (Table 2). In all three FRs, key elements such as (K), (Ca), and (Mg) were present in small concentrations.

**Table 2:** Main chemical elements (mg/L) and physical parameters of the three fire retardants (FRs) evaluated, based on the recommended application ratio of 1:10 (*v*:*v*).

N-Borate 1.5%				N-Phosphate+ 1.5%				N-Phosphate− 2%			
Total Elements		Ions *		Total Elements		Ions		Total Elements		Ions	
N	2662	NO_2_^−^	114	N	2940	NO_2_^−^	<DL	N	507	NO_2_^−^	<DL
P	1232	NO_3_^−^	5.23	P	2937	NO_3_^−^	1428	P	621	NO_3_^−^	613
K	1.0	NH_4_^+^	1039	K	18.6	NH_4_^+^	2296	K	1.8	NH_4_^+^	1674
Ca	<DL	K^+^	7.87	Ca	13.89	K^+^	13.67	Ca	<DL	K^+^	<DL
Mg	0.21	PO_4_^3−^	<DL	Mg	26.96	PO_4_^3−^	1047	Mg	<DL	PO_4_^3−^	34.64
Al	<DL	Ca^2+^	50.36	Al	97.75	Ca^2+^	62.27	Al	<DL	Ca^2+^	203
Fe	<DL	Mg^2+^	44.66	Fe	427	Mg^2+^	33.46	Fe	0.03	Mg^2+^	75
S	13.30	SO_4_^2−^	<DL	S	229	SO_4_^2−^	28	S	0.68	SO_4_^2−^	0.17
B	1953	Na+	49.5	B	4.79	Na+	24.03	B	<DL	Na+	30.5
pH	9.1	Cl^−^	15.14	pH	6.95	Cl^−^	5.69	pH	7.78	Cl^−^	36
DO	4.8	Br^−^	8.6	DO	5.4	Br^−^	48.15	DO	7.0	Br^−^	6.75
EC	2960	F^−^	<DL	EC	11,040	F^−^	15.5	EC	3910	F^−^	<DL
		Li	<DL			Li	<DL			Li	<DL

* The solubilization of cations and anions (mg/L) in the FR solution (FR + distilled water). <DL stands for below detection limit (<0.001 mg/L); DO: Dissolved oxygen (mg/L); EC: Conductivity (μS/cm).

Micronutrients like (Al) and (Fe) were found in N-Phosphate+ FR and were not detected in the other two formulas. The (S) levels in N-Phosphate+ FR were 20 and 200 times higher than in N-Borate FR and N-Phosphate− FR, respectively. Notably, the composition of N-Phosphate− FR was primarily comprised of (N) and (P), rendering it the simplest in terms of composition among the three (Table 2). The total chemical concentration of the FR compounds (100% pure) is displayed in Table S2.

The water-soluble elements in the three FRs revealed the following dominant ions: for N-Borate FR, the predominant ions were NH_4_^+^ > NO_2_^−^ > Ca^2+^ > Na ^+^ > Mg^2+^; for N-Phosphate+ FR, the leading ions were NH_4_^+^ > NO_3_^−^ > PO_4_^3−^> Ca^2+^ >Br^−^ > Mg^2+^, and for N-Phosphate− FR, the primary ions identified were NH_4_^+^ > NO_3_^−^ > Ca^2+^ > Mg^2+^ > Cl^–^ > PO_4_^3−^ (Table 2). The ion composition varied across the three FRs, especially for NH_4_^+^, PO_4_^3−^, SO_4_^2−^. The ion F^−^ was found only in N-Phosphate+ FR. Meanwhile, the cation Li^+^ remained undetected in all three FRs (Table 2).

The levels of available nitrogen (NH_4_^+^, NO_2_^−^, NO_3_^−^) differed among the three FRs, with NH_4_^+^ and NO_3_^−^ found in all three FRs, while NO_2_^−^ was present only in the N-Borate FR (Table 2). Additionally, N-Phosphate+ FR had the highest concentrations of PO_4_^3−^, exceeding those in the N-Phosphate− FR by a factor of thirty. When concentrations of these ions were converted to the highest concentrations of the tests (Table 3 and Table S3), few ions presented relevant concentrations, notably NH_4_^+^ and NO_2_^−^ for N-Borate FR, NH_4_^+^, NO_3_^−^, and PO_4_^3−^ for N-Phosphate+, and NH_4_^+^ for N-Phosphate− FR (Table 3).

Regarding pH values, N-Borate FR was the only one found to be alkaline. N-Phosphate− FR exhibited a neutral pH of 7.35, and N-Phosphate+ FR had a slightly acidic pH of 6.9 (Table 2). These data are consistent with the pH values observed in pure FRs (Table 2 and Table S3). The EC levels were elevated in all three FR solutions, with N-Phosphate+ FR displaying the highest EC (11,040 μS/cm), followed by N-Phosphate− FR (3910 μS/cm) and N-Borate FR (2960 μS/cm). However, during the *C. dubia* tests the EC levels significantly decreased, with N-Borate displaying the highest EC (848 μS/cm), followed by N-Phosphate+ FR (448 μS/cm) and N-Phosphate− FR (256 μS/cm), as presented in Table 3. The DO levels in the three retardant solutions ranged from 7.0 to 4.8 mg/L (Table 2). During the tests, these levels varied from 6.7 to 5.3 mg/L (Table 3).

**Table 3:** Cations and anions (mg/L) from the three fire retardant (FR) solutions and the physical parameters at the highest concentration treatment from *C. dubia* acute tests.

Ions	N-Borate 0.15%	N-Phosphate+ 0.075%	N-Phosphate− 0.02%
F^−^	<DL	0.78	<DL
Cl^−^	1.51	0.28	0.36
NO_2_^−^	11.37	<DL	<DL
Br^−^	0.86	2.41	0.07
NO_3_^−^	0.53	71.24	6.13
PO_4_^3−^	<DL	52.35	0.35
SO_4_^2−^	<DL	1.40	0.002
Li	<DL	<DL	<DL
Na^+^	4.95	1.20	0.30
NH_4_^+^	103.9	114.8	16.74
K^+^	0.79	0.68	<DL
Ca^2+^	5.04	3.11	2.03
Mg^2+^	4.47	1.67	0.75
pH (C0–C5)	7.42–7.31 *	7.67–7.2	7.38–7.68
DO (C0–C5) **	7.1–6.0	6.1–6.7	5.4–5.3
EC (C0–C5)	225–848	187–448	219–256

* Adjusted pH for the test. ** The physical parameters indicated were obtained from the final *C. dubia* tests. C0: control treatment; C5: highest concentration used in the test which corresponds to the respective column. <DL stands for below detection limit (<0.001 mg/L).

### 3.2. Acute Immobilization Tests

**Figure 1:**
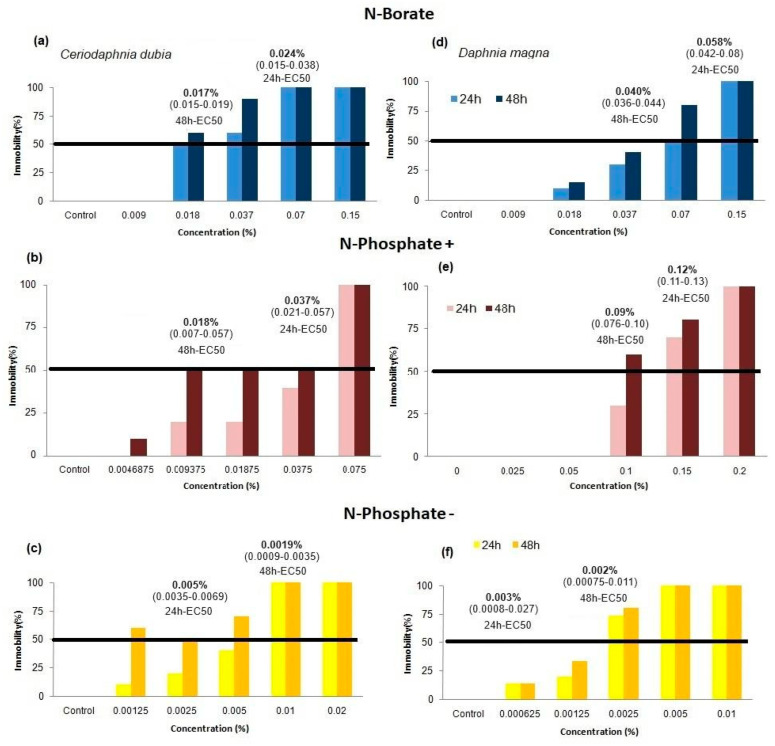
Effect concentration causing immobility in 50% of *C. dubia* (on the left) and *D. magna* (on the right) when exposed to the three different fire retardants (Confidence Interval: 95%): (**a**,**d**) display results for N-Borate FR (a product predominantly composed of N and B), (**b**,**e**) for N-Phosphate+ FR (a product with elevated levels of N and P), and (**c**,**f**) for N-Phosphate− FR (a product primarily containing N and P, albeit at lower concentrations).

During the toxicity testing, the physicochemical parameters of the test solutions remained within the prescribed limits (pH between 7.0 and 7.6, and hardness between 40 and 48 mg/L as CaCO_3_), except for the N-Borate FR, which required pH adjustment (Table 3 and Table S2). The survival rates in the control groups surpassed 80% in all tests (see Figure 1), thereby fulfilling the OECD guidelines for the validity of the tests [31,32].

All three of the FRs tested were shown to affect *C. dubia* and *D. magna* immobility in 24-h and 48-h acute tests. For *C. dubia*, the 24-h EC50 and 48-h EC50 values for N-Borate FR were 0.024% and 0.017%, respectively. For *D. magna*, the corresponding EC50 values for N-Borate FR were 0.06% for 24-h and 0.04% for 48-h. Furthermore, at a dilution of 0.07% N-Borate FR, *C. dubia* showed a 100% immobility rate after 48-h (Figure 1).

Exposure to N-Phosphate+ FR resulted in a 24-h EC50 of 0.037% and a 48-h EC50 of 0.0018% for *C. dubia*. Meanwhile, *D. magna* showed a 24-h EC50 of 0.12% and a 48-h EC50 of 0.09% when exposed to the same FR, indicating again that *C. dubia* has higher sensitivity to N-Phosphate+ FR compared to *D. magna*. As for N-Phosphate− FR, *C. dubia* had a 24-h EC50 of 0.005% and a 48-h EC50 of 0.0019%. *D. magna* had a 24-h EC50 of 0.003% and a 48-h EC50 of 0.0023%, revealing N-Phosphate− FR as the most toxic among the tested FRs, with the 24-h and 48-h EC50 values being closely matched for both species (Figure 1).

## 4. Discussion

Effective wildfire management is essential for preserving environmental, social, and economic assets [33]. However, the use of FRs in actions to fight wildfire must consider their ecological impact. During a megafire in the Pantanal biome, recognized as the world’s largest wetland and located in the states of Mato Grosso and Mato Grosso do Sul, Brazil [34], firefighting operations involved the use of FRs [25]. This application of FRs likely resulted in their dissemination into aquatic environments, raising concerns about possible unexpected effects on these ecosystems.

The toxicity of FRs is determined by their chemical compositions, which are kept confidential [35]. These products contain a wide range of compounds, with inorganic salts being the primary components [15]. While inorganic salts have low toxicity [36], their mixture with other chemicals like thickening agents, surfactants, foam stabilizers, wetting agents, or solvents may lead to larger negative environmental impacts [22].

### 4.1. Potential Impact of the Nutrient Increment on Water Ecosystems

During wildfires, inorganic elements are remobilized and transported by surface water runoff into aquatic systems, leading to increased water turbidity, organic matter, alkalinity, pH, conductivity, and dissolved oxygen depletion [10,37,38]. This post-fire influx can have complex impacts on water quality and aquatic organisms [39,40,41,42]. Elements like Ca and K, typically not considered hazardous, can influence the overall toxicity of ash eluates [43]. Some elements, such as K, S, and B, are highly water-soluble, while others like Ca, Mg, Si, and Fe dissolve more with increased water, and P tends to be insoluble due to precipitation with Ca [44].

The chemical analysis of fire retardants (FRs) in our study revealed a wide range of total and major water-soluble elements that could impair water quality (Table 2 and Table 3). The fire-extinguishing efficacy of FRs is primarily due to ammonium polyphosphate complexes, which can release ammonium (NH_4_^+^) and phosphate (PO_4_^3−^) ions in water [45,46], thereby contributing to stream eutrophication, similar to agricultural and urban runoff [23,47,48]. A previous study pointing to FRs as a source of ammonium phosphate indicated no differences in the growth of the cyanobacterium *Anabaena* when treated with ammonium phosphate compared to the Phos-Chek LC-95 FR [49]. Nitrate primarily harms aquatic animals by converting oxygen-carrying pigments into forms unable to carry oxygen [50,51]. However, nitrate uptake in aquatic animals is more limited than that of NH_4_^+^ and NO_2_^−^, which partially explains nitrate’s lower toxicity [52]. Scott and Crunkilton [53] found the acute toxicity (48-h LC50) of NaNO_3_ was 374 mg NO_3_-N/L for *C. dubia* and 462 mg NO_3_-N/L for *D. magna*. Thus, the LC50 for NO_3_^−^ was much higher than the concentrations observed in our tests (Table 3).

Sulfate toxicity studies reported high 48-h LC50 values for *C. dubia* (48-h LC50 values of 1869 and 4220 mg SO_4_^2−^/L under conditions of low and high-water hardness, respectively), which exceed sulfate concentrations found in the FRs studied [54]. Similarly, KCl toxicity values for aquatic organisms surpass the potassium levels in the FRs, such as the following: 48 h-EC50 = 630 mg/L for *C. dubia,* 48 h-EC50 = 610 mg/L for *D. magna*, and 48 h-EC_50_ = 910 mg/L for the fathead minnow *Pimephales promelas* [55].

Most aquatic organisms require a neutral pH range (6.0 to 8.0) for optimal biodegradation [56]. The initial pH of the evaluated FR solutions varied, with N-Borate FR having the highest pH, necessitating adjustment for use in exposure tests (Table 3 and Table S1). The pH significantly affects metal retention in soils, with lower solubility of metal cations at high- and mid-pH conditions [57]. Increased pH levels can lead to the formation of non-ionized ammonia, which is potentially toxic to aquatic organisms [58].

The three FRs were easily dissolved in the culture medium without creating a separate layer, although the N-Phosphate+ FR was the only which exhibited a tendency to precipitate at the bottom of the beaker. The toxicity of environmental contaminants can be mitigated when they are complexed, precipitated, or adsorbed by inorganic materials and humic acids [59,60]. However, filtering ash solutions may enhance the bioavailability of toxic elements, resulting in increased toxicity to *C. dubia* [12].

N-Phosphate+ FR displayed the highest EC levels, whereas N-Borate FR had the lowest EC levels but the highest pH, which could lead to non-ionized ammonia toxicity (Table 3). Post-fire inputs can decrease fish density due to total dissolved solids and sediment deposition, which can clog fish gills and lead to hypoxia [37,61,62]. In contrast, the EC levels in the assays were higher in N-Borate FR than N-Phosphate+ FR and N-Phosphate− FR (Table 3).

Salinity changes the viscosity of water, affecting the swimming velocity and feeding rate of *D. magna* [63]. The salinity tolerance of freshwater zooplankton depends on both the total amount of dissolved salts and their ionic composition [64]. Increased salinity can decrease the number of moults and delay the first reproduction, as observed in studies with *D. magna* and *D. spinulata* [65,66]. Salt concentrations of 2–4 g/L (~3130–6250 μS/cm) negatively impacted the population growth of *D. pulex* and *Simocephalus vetulus* [67]. The sublethal salinity level (0, 2, 4, 6 and 7 g/L NaCl) with *D. magna* is 5.48 g/L, with some reproduction observed at 7 g/L (~14,000 μS/cm) [68,69]. Although the concentrations used in the test showed EC lower than the levels that cause toxic effects for cladocerans, it is important to highlight that concentrations of FRs below the recommended levels (Table 2) have a high enough EC to cause salinity-induced toxicity.

Ash dissolution tests showed that while only a small portion of ashes dissolve, they can significantly increase pH and reduce dissolved oxygen levels, which can be detrimental to aquatic species [12]. In our tests, the dissolved oxygen was not affected by the FRs (Table 3 and Table S1).

### 4.2. The Toxicity of Evaluated FRs

The three FRs studied exhibited substantial concentrations of NH_4_^+^, with N-Phosphate+ FR showing higher levels than the other two FRs (Table 3). Moreover, N-Phosphate+ FR had the highest concentrations of PO_4_^3−^, exceeding those in N-Phosphate− FR by approximately 20-fold and those in N-Borate FR by approximately 5000-fold (Table 2). N-Borate, on the other hand, shows a high total nitrogen content, which is not present in the form of NH_4_^+^, NO_2_^−^, or NO_3_^−^. This suggests that nitrogen is likely associated with other elements. A similar association can be reported for phosphorus (Table 2).

The descending order of toxicity elicited by the three FRs was as follows: N-Phosphate− FR > N-Borate FR > N-Phosphate+ FR for *D. magna*, and N-Phosphate− FR > N-Borate = N-Phosphate+ FR for *C. dubia* (Figure 1). For *D. magna*, the toxicity of N-Phosphate− FR was found to be 20 times and 50 times greater, respectively, than that of N-Borate and N-Phosphate+ FR (Figure 1). Despite the different chemical compositions of N-Borate and N-Phosphate+ FR (Table 1 and Table 2), their EC50 values were remarkably similar for *C. dubia*, as indicated by the confidence intervals (Table 2 and Figure S1).

Comparing EC50 values for *C. dubia* and *D. magna* should be done cautiously. For example, the hardness of reconstituted water used to culture these test species varies significantly: the hardness for *C. dubia* (40 to 48 mg CaCO_3_/L) is considerably lower than that for *D. magna* (175 to 225 mg CaCO_3_/L). Nonetheless, toxicity test data across species within the genera *Ceriodaphnia* and *Daphnia* have shown consistent similarities across a diverse range of substances [70]. Connors et al. [71] found no evidence indicating differences in sensitivity between *C. dubia* and *D. magna* in both acute and chronic tests. They also demonstrated that the relative sensitivity between these two species is comparable to that observed between *D. magna* and *D. pulex* [71]. If the immobilization of *C. dubia* and *D. magna* induced by FRs has a parallel with the effects seen in natural systems, it is important to note that native tropical species have exhibited greater sensitivity to various compounds compared to non-native temperate species [72,73].

The acute toxicity tests for the three FRs highlighted their immobility effects on two cladoceran species, with N-Phosphate− FR emerging as the most toxic to both species. The 48-h EC50 values were low (0.0018% for *C. dubia* and 0.0023% for *D. magna*), which are four orders of magnitude lower than the recommended application concentration of 20% (Table 1).

The ecotoxicological effects of landfill leachate on *D. magna* approached 1% for the 48-h EC50, demonstrating that the effluent was very toxic [58]. In the context of textile effluents, Garcia et al. [62] reported that the average 48-h EC50 for *D. similis* was 9.28% ± 0.32%, showcasing the toxic nature of these samples.

Apart from direct toxicity of FRs, additional ingredients like corrosion inhibitors could interact synergistically with ammonia, potentially exacerbating the toxicity of FRs [74]. In our study, although N-Phosphate+ FR did not exhibit the highest level of toxicity among the FRs on cladoceran species, it contained the most significant concentration of NH_4_^+^ (Table 3). Meanwhile, N-Phosphate− FR was highly soluble and produced bubbles, which was likely a result of the surfactants in its formula (referenced in Table 1).

Surfactants, by reducing surface tension and inducing foam formation, are considered the main contributors to the toxicity of FRs [75,76,77]. They can disrupt water bodies’ natural biodegradation activities, reduce dissolved oxygen levels, and increase the solubility of organic contaminants, ultimately leading to a decline in water quality [78,79]. Additionally, surfactants can induce harmful effects in aquatic organisms across various trophic levels, including cellular damage, biochemical and physiological disruptions, and reproduction and growth alterations [79,80]. Acute surfactant effects on *D. magna* exposure to linear alkylbenzene sulfonate (LAS) resulted in a 48-h EC50 of 6.31 mg/L [81].

In an assessment of the acute toxicity of ammonia-based fire retardants (FRs), surfactant-based fire-suppressant foams, nitrogenous chemicals, and anionic surfactants (LAS and sodium dodecyl sulfate [SDS]) to juvenile rainbow trout in soft water, Buhl and Hamilton [20] reported that foams generally exhibited greater toxicity than ammonia-based FRs, un-ionized ammonia was the most toxic component (96-h LC50 NH_3_ = 0.125 mg/L for N; 96-h LC50 = 0.79 mg/L for NO_2_-N; 96-h LC50 = 1658 mg/L for NO_3_-N), LAS presented intermediate toxicity (96-h LC50 = 5.0 mg/L), and SDS was the least toxic to rainbow trout in comparison to foams (96-h LC50 = 24.9 mg/L).

In another assessment of the toxicity of nine FRs, *D. magna* showed sensitivity to Phos-Chek WD881C (48-h EC50 = 3 mg/L) and Phos-Chek WD881 (48-h EC50 = 7 mg/L), both of which contain surfactant alpha-olefin sulfonate solution (AOS) in concentrations of approximately 60–80% [82]. Given that previous studies reported the 48-h EC50 of AOS for *D. magna* as 8.7 (7.7 to 9.7) mg/L [83], Anderson and Prosser [82] reported that the observed toxicity could be attributed to the AOS with another component present in these FRs.

The toxic effects of N-Phosphate+ FR might be attributed to its ammonia content, which was noted with other ammonium salt-based FRs tested on *D. magna* [83]. Elevated concentrations of ammonia can induce oxidative stress and disrupt ion regulation in yellow catfish (*Pelteobagrus fulvidraco*) juveniles [84]. Additionally, acute exposure to current-use FRs has been associated with gill damage and decreased survival rates in salmon smolts [18].

In relation to N-Borate FR, this product exhibited elevated levels of (N) and (B), (Table 2). In freshwater environments with a pH range of 6 to 9, the primary form of (B) is unassociated boric acid [85]. Maier and Knight [86] showed that the acute toxicity level of (B) to *D. magna* (48-h LC50) was at 141 mg/L. Additionally, they observed no significant effects of water hardness or sulfate presence on (B) toxicity. In our study, the 48-h EC50 for N-Borate FR was 540 mg/L (Table S4). Thus, our findings underscore the complexity of chemical interactions within mixtures, which do not always correspond to effects predicted from individual substances, highlighting the effects of synergic compounds in mixture composition and/or insufficient information available for understanding the toxicity of newly formulated commercial FRs.

In an acute toxicity assessment conducted by McDonald et al. [75], *D. magna* was exposed to FRs (Fire-Trol GTS-R, Fire-Trol LCG-R, Phos-Chek D75-F), and foam suppressants Phos-Chek WD-881 and Silv-Ex. Of the substances tested, Silv-Ex was identified as the most toxic to daphnids, with a 48-h LC50 of 7 mg/L in both soft and hard waters, whereas Fire-Trol LCG-R exhibited the lowest toxicity, with a 48-h EC50 of 848 mg/L in soft water and 813 mg/L in hard water. In our study, the 48-h LC50 values for *D. magna* were 1328.40 mg/L, 540 mg/L, and 25.48 mg/L for N-Phosphate+ FR, N-Borate FR, and N-Phosphate− FR, respectively (Table S4). For *C. dubia*, the 48-h EC50 values were 267 mg/L, 236 mg/L, and 21 mg/L for N-Phosphate+ FR, N-Borate FR, and N-Phosphate− FR, respectively (Table S4).

The acute toxicity levels for *D. magna* reported in the N-Phosphate− FR data sheet (48-h EC50 > 100 mg/L) differ from the data of this study, which recorded a 48-h EC50 of 25.48 mg/L for *D. magna*. Additionally, the manufacturer’s data sheet lists the EC50 for N-Phosphate− FR for *D. rerio* (96-h EC50 > 100 mg/L) and for the earthworm *E. fetida* (14-day EC50 > 1000 mg/L).

While current Brazilian regulations do not specifically cover the use of FRs for wildfire management [87], manufacturers assert that their products are non-toxic, eco-friendly, and biodegradable (Table 1). They argue their products are likely to present a low risk of toxicity to aquatic species at the concentrations observed in natural environments.

The sensitivity of aquatic organisms to FRs varies significantly across different formulations and species [22,87]. It has been established that *D. magna* is generally more vulnerable to surfactants than other aquatic invertebrates and fish [88]. However, Pane et al. [89] found that *D. magna* was less sensitive to the firefighting surfactant F-500, widely used to combat hydrocarbon spills in freshwater environments, with 24-h EC50 and 48-h EC50 values of 5.17 mg/L and 3.81 mg/L, respectively. This contrasts with larval rainbow trout, which exhibited greater sensitivity (48-h EC50 = 1.41 mg/L and 96-h LC50 = 1.26 mg/L). The toxicity of F-500 FR may be linked to its content of long-chain aliphatic alcohols [89], with an increase in the chain length of these alcohols associated with higher toxicity towards *D. magna* [90].

Zooplankton connect primary producers with predators and significantly influence phytoplankton biomass, affecting both its abundance and species composition [91]. The observed 43% increase in *R. subcapitata* biomass upon exposure to FRs such as Fire-Trol GTS-R, Fire-Trol LCG-R, and Phos-Check D75-F could be attributed to the ammonium and/or phosphorus present in their formulations [92]. On the other hand, Graetz et al. [22] reported that exposure to FRs (Eco-Gel, Thermo-Gel, FireAde, Fire-Brake, Novacool Foam, and F-500) resulted in growth inhibition of *Lemna minor*, and they suggested that these FRs either do not have ammonium and phosphorus or contain another component that negatively affects aquatic plants.

The effects of FRs are an important issue when considering the conservation of aquatic ecosystems amidst wildfire fighting strategies. Discussing ecotoxicological data of FRs could be one way to foster innovations in producing environmentally friendlier alternatives or to possibly replacing FRs with less toxic counterparts with similar efficacy in firefighting.

The range of concentrations used in this study offers insights into the short-term effects of FRs on zooplankton communities and their responses in aquatic systems. This study underscores the need for chemical analyses of other FR compounds, particularly surfactants, and further research on the ecotoxicological impacts of FR and ash runoffs on aquatic biota. Additionally, it is essential to understand the moment when FR concentrations momentarily spike [93], because water flow dynamics in lotic water bodies mitigate the physicochemical effects on water quality from FR applications [20,93].

Finally, we affirm the importance of integrating in vitro assays with field observations and simulation/modeling. Utilizing landscape analysis, pluviometric data, and hydrological studies may raise possibilities for integrated studies, based on the thesis that the observed and estimated FR concentration in water bodies could substantially differ from typical values used in vitro. Furthermore, the residence time of pollutants in streams, rivers, and ponds likely deviates from the conventional 24- or 48-h benchmarks, with concentrations expected to decline markedly within a short period due to high hydrological turnover rates.

Despite the promise of this novel approach, there remains considerable uncertainty regarding both the management of diverse hydrological processes across landscapes and the complex hydrology of water bodies and watersheds. Addressing this challenge is crucial for developing regulatory guidelines for FR usage that balance effective firefighting operations with water resource conservation.

## 5. Conclusions

Wildfires are going to continue to increase in size and severity with climate change, and finding innovative ways to manage them and protect environmental resources will be ever more critical. This study revealed that three commercial FRs impacted the survival of the freshwater cladocera species, *C. dubia* and *D. magna*, at dilution levels three to four orders of magnitude lower than recommended use. The broad range of chemical compositions of the tested FRs resulted in varying levels of toxicity, particularly for N-Phosphate− FR. In addition, *C. dubia* showed greater sensitivity than *D. magna* to N-Borate and N-Phosphate+ FR.

For comprehensive environmental risk assessments, it is crucial to balance the high effectiveness of FRs with minimal risks to the species under projected exposure conditions. In this context, our study provides an understanding of the acute toxic effects of the three FRs for zooplanktonic species. It underscores the importance of a more in-depth investigation into the environmental effects of FRs, including their chemical compositions, degradation rates, and toxic effects on a broader range of organisms across different environmental matrices.

## Figures and Tables

**Table 1:** The formulation, density, and manufacturer-recommended application rates of the three fire retardants (FRs) obtained from the safety data sheets.

Type of Formulation	Known Constituents	Density (g/mL)	Recommended Application Rate (%) *
N-Borate	Organo-mineral fertilizer	1.35	15
N-Phosphate+	Ammonium polyphosphate-based liquid concentrate (80–100%) that contains performance additives (5–10%), attapulgus clay (1–5%) and iron-oxide color (1–5%) as well as ammonium polyphosphate	1.476	15.38
N-Phosphate−	Biodegradable product based on neutralized phosphorus esters, synthetic surfactants, and preservatives; N, P and K used for soil and plants.	1.108	20

* Recommended application to be used for wildfires (*v*/*v*).

## Data Availability

Data presented in this study are available only in this text presented.

## Supplementary Materials

The following supporting information can be downloaded at: https://www.mdpi.com/article/10.3390/toxics12080548/s1. Table S1: *Ceriodaphnia dubia* and *Daphnia magna* physical-chemical parameters of the tests for each fire retardant (N Borate, N-Phosphate+, and N-Phosphate−) initial/final. Table S2: The concentrations of main elements (g/L) from the three fire retardants (FRs) evaluated. Table S3: Cations and anions (mg/L) from the three fire retardant (FR) solutions and the physical parameters at the highest concentration treatment from *D. magna* acute tests. Table S4: Effect concentration in 50% immobility (EC50) for *C. dubia* and *D. magna* when exposed to three distinct fire retardants (FRs), classified by their dominant chemical composition in acute toxicity tests (Confidence Interval: 95%). Figure S1: Box-plots representing 24-h/48-h EC_50_ for *Ceriodaphnia dubia* and *Daphnia magna* of the three FRs studied.
