# Supplementary material for: Acute Toxicity of Commercial Wildfire Retardants to Two Daphniid Species (Ceriodaphnia dubia and Daphnia magna)
Source: Toxics. 2024 Jul 29;12(8):548. doi: 10.3390/toxics12080548 (PMC11360807; doi:10.3390/toxics12080548)
Supplement: Supplementary file 1 [file toxics-12-00548-s001.zip › toxics-3058235-supplementary.pdf]

Supplementary Data

Table S1 *Ceriodaphnia dubia* and *Daphnia magna* physical-chemical parameters of the tests for each fire retardant (N Borate, N-Phosphate+, and N-Phosphate−) initial/final

Ceriodaphnia dubia

Parameters	Concentrations (%) – N-Borate					
	Control	0.009	0.0018	0.0037	0.075	0.15
pH	7.76/ 7.42	7.61/ 7.43	7.60/ 7.45	7.59/ 7.43	7.44/ 7.42	7.38/ 7.31
Dissolved oxygen (mg/L)	9.2./ 7.1	8.6/6.1	8.9/ 6.3	8.9/ 6.2	8.9/ 6.2	8.9/ 6.0
Conductivity (µs/cm)	216/ 225	247/ 249	288/ 281	353/ 392	504/ 534	829/ 848

Parameters	Concentrations (%) - N-Borate (without pH adjustment)					
	Control	0.009	0.0018	0.0037	0.075	0.15
pH	7.26/ 7.9	8.78/ 7.76	- / 8.13	- / 8.44	8.89/ 8.61	- / -
Dissolved oxygen (mg/L)	-/ 5.0	-/ 5.0	-/ 5.0	-/ 5.0	-/ 5.0	-/ 5.0
Conductivity (µs/cm)	-/ 250	- /256	- / 298	- / 354	- / 453	- / -

Parameters	Concentrations (%) - Phosphate+					
	Control	0.002	0.004	0.009	0.0018	0.035
pH	7.32/ 7.67	7.18/ 7.40	7.10/ 7.31	7.11/ 7.21	7.06/ 7.16	7.07/ 7.12
Dissolved oxygen (mg/L)	6.1/ 5.6	6.3/ 6.7	6.3/ 6.2	6.3/ 6.5	6.3/ 6.7	6.4/ 6.7
Conductivity (µs/cm)	181/ 187.1	225/ 228	249/ 263	311/ 319	432/ 448	686/ 706

Parameters	Concentrations (%) - N-Phosphate−					
	Control	0.0012	0.0025	0.005	0.01	0.02
pH	7.31/ 7.38	7.38/ 7.43	7.41/ 7.57	7.52/ 7.61	7.56/ 7.64	7.62/ 7.68
Dissolved oxygen (mg/L)	6.4/ 5.4	5.8/ 5.3	5.8/ 5.4	- / 5.5	- / 5.3	5.6/ 5.3
Conductivity (µs/cm)	177.6/ 219	181.5/ 187.2	187.5/ 188.4	189.2/ 198	216/ 229	248/ 256

Daphnia magna

Parameters	Concentrations (%) – N-Borate (pH adjusted)					
	Control	0.009	0.0018	0.0037	0.075	0.15
pH	7.8/7.4	7.94/8.0				7.82/7.61
Dissolved oxygen (mg/L)	- / -	- / -	- / -	- / -	- / -	- / -
Conductivity	- / 578	- /633	- /695	- /789	- /997	- /1,373

(μs/cm)

Parameters	Concentrations (%) - N-Borate (without pH adjustment)						
	Control	0.0003	0.0006	0.0012	0.0025	0.005	0.01
pH	7.8/7.74	8.37/8	8.51/8.14	8.59/8.14	8.65/8.61	8.69/8.79	8.7/8.97
Dissolved oxygen (mg/L)	- / -	- / -	- / -	- / -	- / -	- / -	- / -
Conductivity (μs/cm)	763/805	753/778	754/776	769/805	793/841	845/891	948/1,000

Parameters	Concentrations (%) - Phosphate+					
	Control	0.0025	0.005	0.01	0.02	0.04
pH	7.77/7.71	7.77/7.52	7.2	7	7	7.06/7
Dissolved oxygen (mg/L)	- / -	- / -	- / -	- / -	- / -	- / -
Conductivity (μs/cm)	622	709	894	1,245	2,16	3,85

Parameters	Concentrations (%) - N-Phosphate−					
	Control	0.000625	0.00125	0.0025	0.005	0.01
pH	8.4/8.06	7.61/8.01	- / -	- / -	- / -	7.63/7.58
Dissolved oxygen (mg/L)	- / -	- / -	- / -	- / -	- / -	- / -
Conductivity (μs/cm)	565 /572	560/580	- / -	- / -	600/610	750/765

Table S2 - The concentrations of main elements (g/L) from the three fire retardants (FRs) evaluated.

Chemical element	N-Borate	N-Phosphate^{+}	N-Phosphate−
**N**	177.4	196	25.35
**P**	0.08	196	31.06
**K**	0.07	1.24	0.09
**Ca**	<DL	0.93	<DL
**Mg**	0.014	1.80	<DL
**Al**	<DL	6.51	<DL
**Fe**	<DL	28.44	<DL
**S**	0.90	15.30	0.034
**B**	130.2	0.32	<DL
**pH**	8.44	6.90	7.35

<DL stands for below detection limit (<0.001 mg/L).

Table S3- Cations and anions (mg/L) from the three fire retardant (FR) solutions and the physical parameters at the highest concentration treatment from *D. magna* acute tests.

Ions	N-Borate 0.15%	N-Phosphate$^+$ 0.2%	N-Phosphate− 0.01%
**F^-**	<DL	2.07	<DL
**Cl^-**	1.51	0.76	0.18
**NO_2^-**	11.37	<DL	<DL
**Br^-**	0.86	6.42	0.034
**NO_3^-**	0.53	190	3.07
**PO_4^{3-}**	<DL	139.6	0.17
**SO_4^{2-}**	<DL	3.74	<DL
**Li**	<DL	<DL	<DL
**Na^+**	4.95	3.20	0.15
**NH_4^+**	104	306	8.37
**K^+**	0.79	1.83	<DL
**Ca^{2+}**	5.04	8.30	1.02
**Mg^{2+}**	4.47	4.46	0.37

<DL stands for below detection limit (<0.001 mg/L).

Table S4 - Effect concentration in 50% immobility (EC50) for *C. dubia* and *D. magna* when exposed to three distinct fire retardants (FRs), classified by their dominant chemical composition in acute toxicity tests (Confidence Interval: 95%).

Fire Retardant type	24h-EC_{50}<br>(%)<br>(ppm)<br>(g/L)*	48h-EC_{50}<br>(%)<br>(ppm)<br>(g/L)	Concentrations tested<br>(%)<br>(ppm)<br>(g/L)
	Ceriodaphnia dubia		
**N-Borate**	0.02409 (0.01542 - 0.03823)<br>240.9 (154.2 - 382.3)<br>0.325 (0.21 – 0.52)	0.01749 (0.01561 - 0.01959)<br>174.9 (156.1 - 195.9)<br>0.236 (0.21 – 0.26)	0 - 0.15<br>0 – 1,500<br>0 – 2.02
**N-Phosphate$^+$**	0.037 (0.02104 - 0.05747)<br>370 (210.4 - 574.7)<br>0.545 (0.31 – 0.846)	0.01811 (0.007068 - 0.05783)<br>181.1 (70.68 – 578.3)<br>0.267 (0.104 – 0.853)	0 - 0.075<br>0 - 750<br>0 – 1.1
**N-Phosphate−**	0.005102 (0.003465 - 0.006897)<br>51 (34.65 - 69)<br>0.056 (0.038 - 0.076)	0.001893 (0.000985 - 0.003506)<br>18.93 (9.85 - 35.06)<br>0.021 (0.011 - 0.039)	0 - 0.02<br>0 - 200<br>0 – 0.22
	Daphnia magna		
**N-Borate**	0.058 (0.042 – 0.080)<br>584.4 (418.2 - 805)<br>0.789 (0.564 – 1.087)	0.040 (0.036 – 0.044)<br>400 (560 - 444)<br>0.54 (0.485 – 0.60)	0 - 0.15<br>0 – 1,500<br>0 – 2.02
**N-Phosphate$^+$**	0.12 (0.11 - 0.13)<br>1,203 (1,072 – 1,335)<br>1.776 (1.58 – 1.97)	0.09 (0.076 – 0.10)<br>886 (761 -1,015)<br>1.33 (1.12 – 1.49)	0 - 0.02<br>0 – 200<br>0 – 0.295
**N-Phosphate−**	0.0030 (0.00081 - 0.027)<br>30.25 (8.10 - 271.8)<br>0.033 (0.009 – 0.30)	0.0023 (0.00075 – 0.011)<br>23.37 (7.50 - 113.4)<br>0.0254 (0.0083 – 0.1218)	0 - 0.01<br>0 – 100<br>0 – 0.11

*Calculated based on the density of each FR, as showed in Table 1.

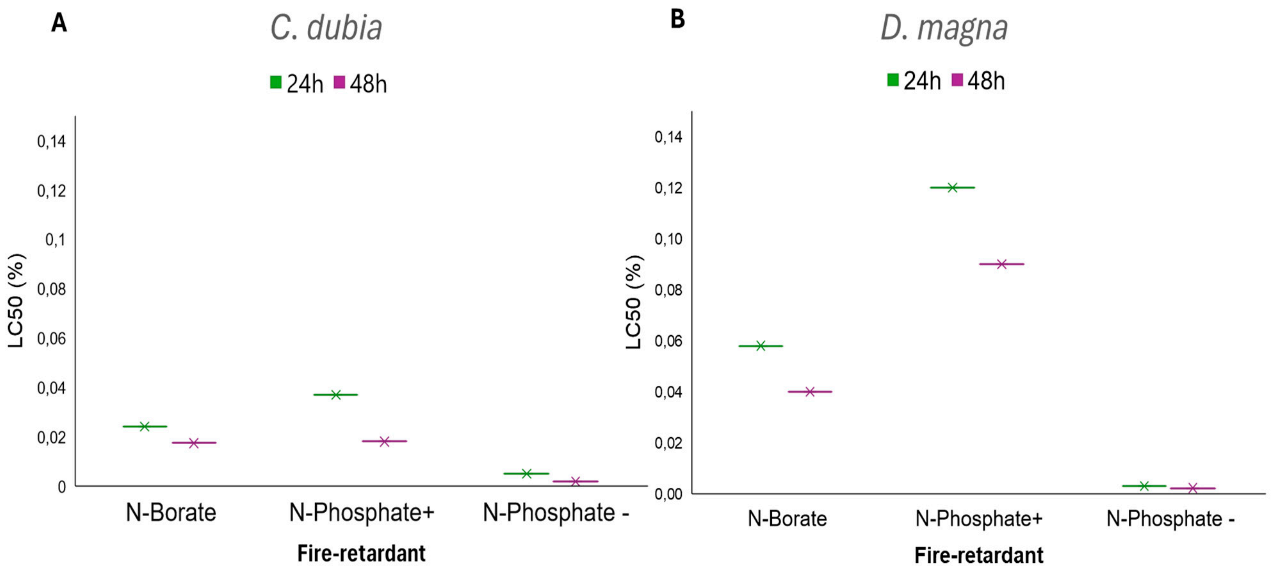

Figure S1. Box-plots representing 24-h/48-h EC_{50} for *Ceriodaphnia dubia* and *Daphnia magna* of the three FRs studied.
